# Male Gender and Low Education with Poor Mental Health Literacy: A Population-based Study

**DOI:** 10.2188/jea.17.114

**Published:** 2007-07-18

**Authors:** Yoshihiro Kaneko, Yutaka Motohashi

**Affiliations:** 1 Department of Public Health, Akita University School of Medicine.

**Keywords:** Mental Health, Learning, Sex, Education, Depression, Suicide

## Abstract

**BACKGROUND:**

Both male gender and low socioeconomic status have been related to depression and suicide, but their possible relationship to mental health literacy remains uncertain. The objectives of this study were to assess the level of mental health literacy in rural communities in Japan and to examine related factors.

**METHODS:**

A population-based cross-sectional study using a questionnaire was conducted. Response rate was 88.2% from 8163 residents aged 30-69 years. The relationships between mental health literacy (including cognition of depression, attitude toward depression, and acceptance of suicide) and demographics, socioeconomic status, and the severity of depression were assessed by logistic regression analysis.

**RESULTS:**

Of the respondents, 25.2% showed an inadequate cognition of depression, 12.5% showed an inadequate attitude toward depression, and 13.1% showed an acceptance of suicide. Of the complete respondents (65.5%), an inadequate cognition of depression was associated with being male (adjusted odds ratio=1.93, 95% confidence interval: 1.68-2.22), advanced age (2.18, 1.58-3.00), and a lower level of education (1.95, 1.34-2.86); an inadequate attitude toward depression was associated with being male (2.18, 1.82-2.61),a lower education (2.34, 1.38-3.97), and the severity of depression (2.26, 1.54-3.32); and an acceptance of suicide was associated with being male (1.33, 1.13-1.58) and the severity of depression (5.77, 4.20-7.93).

**CONCLUSIONS:**

Poor mental health literacy related strongly to male gender and a low level of education. According to our results, poor mental health literacy may possibly be a factor contributing to male vulnerability to suicide.

Suicide has become a pressing public health issue. In Japan, it is the sixth leading cause of death; among males who are middle age or younger, it is the number one cause of death. In many cases, suicide and suicidal behaviour are affected by mental distress.^1^ Depression is one of the important risk factors for suicide. A study of the burden of disease worldwide predicted that major depression will become the second most pervasive disease by 2020.^2^ Unfortunately, the need to treat depression is not well recognized by the general population. Patients with depression have long felt a social stigma associated with their condition, and thatfeeling reduces the likelihood that they will seek treatment.^3^ In Japan, the percentage of people suffering from mental disorders who receive medical treatment has been low historically.^4^ An improper understanding of and ineffective coping with depression in the general population may lead to poor consulting behaviour and an exacerbation of depression, and ultimately to a greater number of suicides.

While the number of suicides is much smaller than the number of depressed patients, more than two-thirds of suicides occur on the first attempt.^5^ Because depressed patients tend to be unable to engage in healthy behaviour due to their symptoms (such as psychomotor retardation), there is a need for adequate social support that will allow them to access proper health care services. Health literacy, including proper cognition and social skills that promote good health,^6^ may facilitate community empowerment that can aid depressed people as well as improve their personal skills. Thus, for suicide prevention, it is worthwhile for people to develop coping skills for depression and suicide ideation. Jorm et al^7^ defined mental health literacy as knowledge and beliefs about mental disorders that aid in their recognition, management, and prevention. Poor mental health literacy may constitute a considerable impediment to the recognition and management of depression.^8^ The authors of that study also suggested that improving mental health literacy for depression reduced the rate of suicide.^9^

Low socioeconomic status (SES) is related to depression^10^^,^^11^ and suicide.^12^^-^^15^ In this regard, the lower socioeconomic groups, the least educated, and those with children showed negative attitudes toward the mentally ill when it comes to setting up mental health service facilities.^16^^,^^17^ In Japan, as in many Western countries, the suicide mortality rate is higher in males than in females.^18^ All of those factors might be affected by the level of mental health literacy, but the relationship remains uncertain.

In the prolonged economic recession of the 1990s, the suicide rate in Japan became the highest among all developed countries. Health education designed to raise awareness of suicide and to facilitate community action was clearly needed. In order to establish effective suicide prevention measures, an assessment of mental health literacy is necessary. However, the level of mental health literacy in Japan had still not been sufficiently evaluated.^19^^,^^20^ Therefore, we conducted a study to evaluate mental health literacy in specific rural communities. Mental health literacy was assessed based on the cognition of depression, the attitude toward depression, and the acceptance of suicide.

Thus, the objectives of this study were to assess the level of mental health literacy in rural communities in Japan and to examine related factors.

## METHODS

### Setting

The survey area consisted of two neighbouring towns in a rural part of Akita Prefecture, which is located in northern Japan. Annual suicide mortality rates in Japan and Akita Prefecture in 2002 were, respectively, 23.3 (male 35.2, female 12.8) and 42.1 (male 65.7, female 20.7) per 100,000 population. Since 1995, Akita Prefecture has recorded the highest suicide mortality rate in Japan. The average suicide mortality rate of the two surveyed towns during 2000 and 2002 was 60.7 per 100,000, markedly higher than the overall rate for Akita Prefecture. During this period, male and female suicide mortality rates in the survey area were, respectively, 68.1 and 54.1 per 100,000. The total area of the two towns at the time of the survey was 190km^2^. Half the area in each town was mountainous; 57.8% was forest; and 20.2% wasfarmland. The populations of the two towns in 2002 were 8,540 and 7,988, respectively.

### Participants

A questionnaire survey on depression and SES variables was conducted, by complete enumeration, from July through September 2003. The target population was 8,163 residents and included those aged 30-69 years while excluding inpatients and institutional residents. A questionnaire was delivered to each household by community volunteers or municipal employees, who two weeks later returned to collect them. A total of 7,202 people (88.2%) responded to the questionnaire.

### Questionnaire

The questionnaire included a vignette that contained classical features depicting depression and was designed to assess mental health literacy. This vignette was referred to in Jorm et al^7^ and was revised for the Japanese setting. The questionnaire read as follows: "Please answer based on the assumption that someone with whom you are familiar (a family member or friend) manifests the following symptoms: The person has, over the last few weeks, been having trouble sleeping, has a poor appetite, and has lost weight. The person has lost vigour and cannot focus on work. When you talk to this person, the answers you receive are minimal and pessimistic." Respondents were asked whether the conduct was caused by mental illness, physical illness, the imagination of the person, or something unknown. They were also asked whether the best thing they could do for the person would be to recommend that the person see a physician, to listen to the person talk about his or her problem, to encourage the person, or to leave the person alone. The acceptance of suicide, as a concept, meant whether or not suicide was inevitable.

We used age and sex as demographic variables. Educational background and current occupational status were used as SES variables. Educational background was classified into four levels: compulsory education (9 years); high school graduate (12 years); junior college graduate/vocational school graduate (14 years); university graduate or higher (16+ years). Occupational status was classified into four categories: non-manual worker (manager included); manual worker; self-employed (farmer included); and unemployed/others.

Severity of depression was assessed using Zung's self-depression scale (SDS).^21^ Respondents were classified into four levels: normal (a raw score of less than 40); mild (40-47); moderate (48-55); and severe (56+).

Respondents were allowed the option to fill in their names and addresses. However, it was made clear to each participant that such personal information would only be used to provide the depression score if requested by the respondent. Thus, we could not analyze the difference between anonymous and non-anonymous participants. The anonymous rate was 52.8% of total respondents.

### Statistical Analysis

The characteristics and the level of mental health literacy were summarized for the total respondents and the complete respondents. The Pearson χ^2^ test and Kendall's τ test were used to compare the characteristics and the level of mental health literacy between the complete respondents and the non-complete respondents. To identify factors related to mental health literacy, responses regarding the cognition of and the attitude toward depression were recorded. The cognition of depression was classified into two groups: a choice of "mental illness" meant an adequate cognition, while the other three choices indicated an inadequate cognition. The attitude toward depression was classified into two groups: recommending that the individual see a physician and listening to the person talk were indications of an adequate attitude, while offering encouragement and leaving the person alone were inadequate. Logistic regression analysis was conducted to evaluate which factors were related to an inadequate cognition of depression, an inadequate attitude toward depression, and the acceptance of suicide. All statistical analysis was performed using SPSS^®^ 11.0 software (SPSS Inc.).

### Ethics

This survey was approved by the Ethics Committee of the School of Medicine at Akita University.

## RESULTS

The characteristics of the respondents are presented in Table 1. The unemployed/others category accounted for 23.3%, which included retired persons and full-time housewives. Regarding the severity of depression, 18.9% of the total respondents had moderate depression and 4.5% had severe depression. Of 8,163 community residents, 5,346 (65.5%) gave complete answers for all variables included in the logistic regression analysis (see Table 1, second column). Males (Pearson χ^2^=21.3), younger people (Kendall's τ = −0.20), those with a higher level of education (Kendall's τ = 0.20), and those employed as non-manual and manual workers (Pearson χ^2^=111.2) had significantly higher completion rates than others (p<0.01).

**Table 1.** Characteristics of respondents included in the sample analyzed (%).*

Items	Respondents	Sample analyzed
Gender	(n=6900)	(n=5346)
Male	47.6	49.1
Female	52.4	50.9

Age (year)	(n=6756)	
30-34	8.3	9.6
35-39	8.9	10.0
40-44	12.2	13.6
45-49	15.2	16.3
50-54	17.1	17.2
55-59	12.4	11.9
60-64	12.3	10.8
65-69	13.5	10.5

Educational background	(n=6900)	
Compulsory education	33.1	27.5
High school graduate	50.7	54.2
Junior college graduate/vocational school graduate	12.5	13.8
University graduate or higher	3.7	4.4

Occupational status	(n=6711)	
Non-manual	18.3	19.9
Manual	34.6	36.1
Self-employed	23.8	22.4
Unemployed/others	23.3	21.5

Depression severity	(n=5985)	
Normal	41.6	41.7
Mild	35.0	35.1
Moderate	18.9	18.9
Severe	4.5	4.4

* : Among the 8163 residents, 5346 (sample analyzed) gave complete answers for all variables included in the logistic regression analyses.

The results of mental health literacy measurement were as follows. In the cognition of depression category, 74.8% of respondents selected an adequate answer, citing mental illness, while 25.2% selected an inadequate answer — i.e. physical illness (9.3%), the depressed person's imagination (3.9%), and unknowncause (12.0%). Sample percentages for the logistic regression analysis were 76.8%, 8.6%, 3.6%, and 11.1%, respectively. Regarding the attitude toward depression, the majority of respondents selected an adequate answer — i.e. recommend that the person see a physician (34.3%) or listen to the person talk about his or her problem (53.2%). The remaining 12.5% selected an inadequate answer — i.e. encourage the person (5.3%) or leave the person alone (7.2%). Sample percentages analyzed were 33.5%, 54.5%, 4.9%, and 7.1%, respectively. Of the total respondents and of the sample analyzed, the percentages of those indicating an acceptance of suicide were 13.1% and 13.3%, respectively. In the sample analyzed, the percentages of respondents with an inadequate cognition of depression (23.2%) and an inadequate attitude toward depression (12.0%) were significantly lower than those of non-complete respondents (Pearson χ^2^=8.6, 59.1, p<0.01).

The results of logistic regression analysis are shown in Table 2. An inadequate cognition of depression was associated with male gender, advanced age, and a lower level of education. An inadequate attitude toward depression was associated with male gender, a lower level of education, being a manual worker, and the severity of depression. An acceptance of suicide was associated with being male, self-employed, unemployed/others, and the severity of depression.

**Table 2.** Odds ratios (ORs) with 95% confidence intervals (CIs) for the poor mental health literacy by the multiple logistic regression.*

Items	d.f.	Inadequate cognition of depression^†^		Inadequate attitude toward depression^‡^		Acceptance of suicide	
		23.2% (1239/5346)		12.0% (639 / 5346)		13.3% (710 / 5346)	
		
		p	OR (95% CI)	p	OR (95% CI)	p	OR (95% CI)
Gender							
Male	1	<0.001	1.93 (1.68-2.22)	<0.001	2.18 (1.82-2.61)	<0.01	1.33 (1.13-1.58)
Female			Reference		Reference		Reference

Age (year)	7	<0.001		0.05		0.17	
30-34			Reference		Reference		Reference
35-39	1	0.31	0.84 (0.61-1.17)	0.02	0.61 (0.41-0.91)	0.29	0.83 (0.59-1.17)
40-44	1	0.63	1.08 (0.80-1.45)	0.48	0.89 (0.63-1.25)	0.04	0.71 (0.51-0.99)
45-49	1	0.62	0.93 (0.70-1.24)	0.13	0.77 (0.55-1.08)	0.18	0.81 (0.59-1.11)
50-54	1	0.13	1.24 (0.94-1.65)	0.02	0.67 (0.48-0.95)	0.16	0.80 (0.58-1.09)
55-59	1	0.05	1.35 (1.00-1.83)	0.24	0.80 (0.55-1.16)	0.19	0.79 (0.56-1.12)
60-64	1	<0.001	1.79 (1.31-2.44)	0.06	0.69 (0.46-1.02)	0.10	0.73 (0.50-1.06)
65-69	1	<0.001	2.18 (1.58-3.00)	0.96	0.99 (0.67-1.47)	<0.01	0.54 (0.36-0.81)

Educational background	3	<0.001		<0.001		0.14	
Compulsory education	1	<0.01	1.95 (1.34-2.86)	<0.01	2.34 (1.38-3.97)	0.06	1.62 (0.99-2.66)
High-school graduate	1	0.09	1.37 (0.95-1.97)	0.08	1.57 (0.95-2.60)	0.25	1.31 (0.82-2.09)
Junior college graduate/vocational school graduate	1	0.24	1.27 (0.85-1.90)	0.38	1.29 (0.74-2.24)	0.29	1.31 (0.79-2.17)
University graduate			Reference		Reference		Reference

Occupational status	3	0.41		0.02		0.07	
Non-manual			Reference		Reference		Reference
Manual	1	0.33	1.10 (0.91-1.34)	<0.01	1.48 (1.15-1.91)	0.41	1.11 (0.87-1.41)
Self-employed	1	0.45	1.09 (0.88-1.35)	0.21	1.21 (0.90-1.62)	0.02	1.37 (1.05-1.80)
Unemployed/others	1	0.10	1.21 (0.97-1.52)	0.11	1.29 (0.95-1.75)	0.04	1.33 (1.01-1.76)

Depression severity	3	0.06		<0.001		<0.001	
Normal			Reference		Reference		Reference
Mild	1	0.04	1.17 (1.01-1.36)	<0.001	1.51 (1.23-1.84)	<0.001	1.56 (1.27-1.92)
Moderate	1	0.01	1.26 (1.05-1.52)	<0.001	1.87 (1.48-2.35)	<0.001	2.86 (2.30-3.56)
Severe	1	0.55	1.11 (0.79-1.57)	<0.001	2.26 (1.54-3.32)	<0.001	5.77 (4.20-7.93)

*: Multiple logistic regression models were adjusted for all items listed in the Table.

†: Cause of features: physical disorder, only imagination and unknown

‡: The best care of the suffering individual: encouragement and leave alone

## DISCUSSION

This study assessed the level of mental health literacy and related factors in rural communities. Over three-fourths of the respondents had an adequate cognition of and an adequate attitude toward the depression depicted in the vignette. One-eighth of the respondents indicated that suicide was inevitable. In a recent Australian study, recognition of depression among the general population showed similar results — i.e. three-fourths of the respondents identified symptoms of depression as a mental disorder (mainly depression).^9^ The prevalence of a suicide-permissive attitude was slightly lower than in a recent study in a neighbouring area of Japan.^19^ In addition, we found that poor mental health literacy was significantly associated with being male, being elderly, and having a lower level of education.

The poor mental health literacy of males was consistent with the suicide gender difference. In regard to suicide mortality, some previous reports have discussed male vulnerability to suicide as being caused by the cultural script pertaining to gender-roles or the confusion resulting from recent gender-role conflicts.^22^^,^^23^ The prevailing gender-role for males — i.e. the traditional masculine stereotype — may cause a reluctance on the part of men to seek help for their depression and result in poor mental health literacy generally among the male sex. Poor mental health literacy may lead to inadequate coping with depression and/or suicide ideation. According to our results, poor mental health literacy may possibly be a factor contributing to male vulnerability to suicide.

Poor mental health literacy showed a strong association with educational level, after adjusting for age and sex. This seemed to be explained by a lack of learning ability on the part of those with a low education rather than a lack of opportunity because there is ample opportunity for every adult in Japan to obtain education regarding mental health, irrespective of one's educational background. Thus, it may depend on the lack of social skills of those with a low education. Gunnell et al^24^ suggested that an association between lower intelligence and the risk of suicide may possibly be due to the importance of cognitive ability in either the aetiology of serious mental disorders or in an individual's capacity to solve problems while going though an acute life crisis or suffering from mental illness. The average amount of education in Japan is not equal for each generation, and it is necessary to deal carefullywith the vulnerability produced by a lower education when implementing community efforts to prevent depression and suicide.

Even after adjusting for educational background, the cognition of depression grew increasingly inadequate with age. The older respondents had few opportunities to acquire health education regarding depression in their youth. This may impair the ability of the elderly to seek help when they need it. Indeed, middle-aged and elderly men in Japan are recognized as a high-risk group for suicide.

It is clear that the severity of depression is related to a poor attitude toward depression and an acceptance of suicide. The attitudes toward depression that we evaluated in this study were related to the help-seeking behaviour of depressed people. A depressed person may become listless and not choose to take a supportive attitude toward another depressed person. Relations between depression severity and suicide ideation were consistent with those of other studies.^25^^,^^26^ Concerning health promotion aimed at suicide prevention, the strong correlation between depression severity and the acceptance of suicide should be addressed publicly in order to raise awareness of suicide. This may facilitate a supportive attitude among the population in confronting depression and suicide ideation.

Our analysis did not show consistent results in terms of current occupational status. Regarding occupation, education stratified analysis showed inconsistent results (data not shown). An interaction model between education and occupation also did not show obvious confounding (data not shown). Relations between mental health literacy and current occupation were uncertain.

Factors related to mental health literacy as revealed by this study suggested that health education must be planned to fit with the prevailing level of mental health literacy, especially in high risk groups. In terms of heath promotion, it is important to raise the level of mental health literacy broadly throughout the community, which will lead to more effective suicide prevention through an improvement of the supportive environment for patients with depression and other mental disorders.

This assessment of mental health literacy used a vignette that focused on responses to a series of symptoms and not on recognizing different mental disorders. Therefore, our results were practical and were not affected by any stigma that might attach to the naming of a specific mental disorder. Mental health literacy may serve as an assessment tool for a suicide prevention program. However, literacy regarding other mental diseases related to suicide, such as alcohol abuse and schizophrenia, was not examined in our current study. In future research, it will be necessary to improve the precision of mental health literacy in this regard.

There were several limitations to this study. First, poor mental health literacy as a cause of suicide was not clarified by this cross-sectional study. Thus, it will be necessary to evaluate whether an improvement in the community's mental health literacy leads to a decrease in the suicide rate. Second, the differences in the levels of mental health literacy between the sample analyzed and the non-complete respondents may affect our results. The differences were mainly due to age and educational background. Because low completion rates were observed among the elderly and because those with a lower level of education had poor mental health literacy, the sample analyzed of those groups had superior mental health literacy than the non-complete respondents. If the non-complete respondents were included in the regression analysis, the ratios for the elderly and those with a lower level of education might have been increased. We must consider the effect of using complete respondents. Third, this study was conducted in rural communities in Japan. Further research will be required in urban areas with different community relationship patterns and SES distributions.

In conclusion, poor mental health literacy is strongly related to male gender and a lower level of education. Our results provide new insight into the gender divergence between depression and suicide. According to our results, poor mental health literacy may possibly be a factor contributing to male vulnerability to suicide. Therefore, a suicide prevention program aimed at improving mental health literacy should take into account the characteristics of males and those with a lower level of education.
